# Foscarnet treatment of cytomegalovirus infection in haploidentical or unrelated donor transplants

**DOI:** 10.1038/s41409-018-0200-y

**Published:** 2018-05-24

**Authors:** Elisabetta Metafuni, Patrizia Chiusolo, Simona Sica, Luca Laurenti, Stefania Bregante, Maria Teresa Van Lint, Alida Dominietto, Emanuele Angelucci, Andrea Bacigalupo

**Affiliations:** 1 Hematology Department, Fondazione Policlinico Universitario Agostino Gemelli, Universita’ Cattolica deI Sacro Cuore, Rome, Italy; 2 Hematology Department, IRCSS Azienda Ospedaliera Universitaria San Martino IST, Genoa, Italy

## Abstract

We studied 97 patients who developed cytomegalovirus (CMV) viremia following an allogeneic hemopoietic stem cell transplant (HSCT) between 2010 and 2015, treated with foscarnet, with the aim of assessing efficacy and safety. The donor was unrelated in 30 patients (UD) and a family HLA-haploidentical donor (HAPLO) in 67 patients: the former (UD) received a prophylaxis for graft-versus-host disease (GvHD), based on antithymocyte globulin (ATG); the latter (HAPLO) received GvHD prophylaxis, based on post-transplant cyclophosphamide (PT-CY). Renal and hematological toxicity were defined according to NCI-CTCAE4 criteria. In univariate analysis, CMV response was 84% in HAPLO vs 59% in UD grafts (*p* = 0.01) and 90 vs 66% (*p* = 0.02) for patients with a CMV viral load within or over the median value. In multivariate analysis, the CMV viral load was the strongest predictor of response to foscarnet (*p* = 0.02), followed by donor type (*p* = 0.06). Renal impairment developed in 14% of the patients. Overall survival was 69%:, advanced phase at transplant (*p* = 0.01) and ATG-based regimens (*p* = 0.02), were  the only two predicting factor. In conclusion, CMV response to foscarnet treatment is predicted by a lower CMV load and GvHD prophylaxis. Renal toxicity of foscarnet is not a limiting factor.

## Introduction

Cytomegalovirus (CMV) infection is one of the most frequent complications after allogeneic hematopoietic stem cell transplantation (HSCT), and occurs in up to 80% of CMV-seropositive patients [1]. The main risk factor for CMV infection is the serological status of both the donor and the recipient [2, 3], but also the use of high-dose corticosteroids, T-cell depletion, acute, and chronic graft-versus-host disease (GVHD), and the use of a donor other than an HLA identical sibling [4]. CMV infection is defined as virus isolation or detection of viral proteins or nucleic acids in any body fluid or tissue specimen [5]. The detection of the CMV pp65 in peripheral blood leukocytes (CMV antigenemia) is a semiquantitative method, indicating the ongoing CMV viremia in transplanted patient [6, 7]. On the other hand, quantitative polymerase chain reaction (qPCR) is the most sensitive method for detecting CMV viremia [8], with a good predictive power for CMV disease in HSCT recipients [9]. Patients are treated at the time CMV infection is diagnosed, to prevent CMV disease, and this is referred to as preemptive treatment [1].

Several drugs are now available for preemptive treatment of CMV infections, and among these, ganciclovir and foscarnet have a long-lasting proof of efficacy [10, 11]. A randomized study in 110 patients with CMV infection compared foscarnet with ganciclovir, the primary end point being survival without CMV disease or death. At 180 days [12], the event-free survival was 66 vs 73% for patients treated with foscarnet or ganciclovir (*p* = 0.6). Another randomized study reported a faster clearance of CMV antigenemia in the foscarnet group, as compared to ganciclovir group, with an overall transplant-related mortality (TRM) of 18%, with no difference in response or mortality in the two groups [13].

However, despite proven comparable efficacy of foscarnet over ganciclovir, ganciclovir is the drug of choice in first line preemptive therapy for CMV, unless the patient is cytopenic, whereas foscarnet is considered to be too nephrotoxic for standard-of-care clinical practice. We thus designed this retrospective study to assess the toxicity profile and efficacy of foscarnet, for alternative donor transplants, known to be at higher risk of CMV infections [4]. We were interested in comparing two different transplant platforms: a conventional unrelated donor graft (UD) with antithymocyte globulin (ATG), based GvHD prophylaxis, and the HLA family haploidentical (HAPLO) grafts with post-transplant cyclophopshamide (PT-CY) prophylaxis, a rapidly growing transplant platform [14].

## Materials and methods

### Patients and transplant characteristics

The study is a retrospective observational analysis on the preemptive use of foscarnet for CMV infection after alternative donor HSCT performed in two transplant units, IRCCS San Martino in Genova and Fondazione Policlinico Universitario Agostino Gemelli in Rome. The aim of the study was to access the efficacy and safety of foscarnet. The study was approved by the IRB of Istituto di Ematologia, Fondazione Policlinico Universitario A. Gemelli in Rome. Patients’ data were retrieved from a prospectively collected computerized database and charts review. The inclusion criteria were as follows: first allogeneic transplants in adult patients who experienced CMV infection after an alternative donor HSCT, performed between 2010 and 2015, who had received foscarnet as preemptive treatment. According to this inclusion criteria, 97 patients were eligible for this study. The underlying disease of these patients was aplastic anemia (AA) (*n* = 4), Hodgkin lymphoma (*n* = 6), non-Hodgkin lymphoma (*n* = 6), acute myeloid leukemia (*n* = 38), primary myelofibrosis (*n* = 14), acute lymphoblastic leukemia (*n* = 13), myeloproliferative disease (*n* = 1), chronic lymphocytic leukemia (*n* = 3), chronic myeloid leukemia (*n* = 3), multiple myeloma (*n* = 1), and myelodysplastic syndrome (*n* = 8). The stem cell source was bone marrow (BM) in 74 cases, peripheral blood (PB) in 20 cases, and cord blood (CB) in three cases. The median dose of CD34+ was 3.1 × 10^6^/Kg (range 1.3–6.5) for BM, 6 × 10^6^/Kg (range 4.3–15.1) for PB, and 3.1 × 10^5^/Kg (range 2.0–5.1) for CB. All patients received acyclovir throughout conditioning and during the first month post-transplant. CMV serostatus was available for 91 patients: it was positive in both donor and recipient (+/+) in 43 pairs (47%), in the recipient only (−/+) in 38 pairs (42%), in the donor only in six pairs (+/−) (7%), and negative in both patient and donor (−/−) in four pairs (4%). Acute GvHD was diagnosed, according to Gluksberg criteria [15]. Chronic GvHD was classified as absent, mild, moderate, or severe, in keeping with the NIH criteria [16].

Clinical characteristics are shown in Table 1, stratified according to donor type: UD or family mismatched (HAPLO). The two groups were comparable, for recipient and donor characteristics. GvHD prophylaxis in UD grafts consisted of cyclosporine A (CSA), methotrexate, and ATG (7.5 mg/kg) (Thymoglobulin Sanofi, France) [18]; for patients receiving HAPLO grafts, prophylaxis was high dose PT-CY on day +3 and +5, CSA and mycophenolate from day 0 [19].

**Table 1:** Clinical characteristics of patients receiving foscarnet

Number of patients	UD	HAPLO	*p*-value
	30	67	
Patients age	48 (19–63)	51 (17–74)	0.9
Center A/B	18/12	64/3	0.01
AML/ALL vs others	15/15	36/31	0.7
Patients gender M/F	17/13	41/26	0.6
Donors age	30 (0–48)	34 (15–63)	0.1
Donor gender M/F	23/7	41/26	0.1
Stem cell source (PB/BM/CB)	20/7/3	0/67/0	–
Donor serostatus pos/neg	11/19	38/23	0.02
Recipient serostatus pos/neg	28/2	53/8	0.35
Days to PMN 0.5 × 10^9^/L	17 (9–34)	18 (14–50)	0.7
aGvHD^a^	19 (63%)	45 (67%)	0.7
Early phase (CR1+CR2)	19 (63%)	39 (71%)	0.4
MA conditioning#	26 (87%)	59 (88%)	0.8
*GvHD prophylaxis*			
ATG+CyA+MTX	30 (100%)		
PT-CY+CyA+MMF		67 (100%)	0.001

^a^aGvHD at the moment of CMV infection

Center A: Genoa; Center B: Rome

*UD* unrelated donor, *HAPLO* family HLA haploidentical donors, *AML* acute myeloid leukemia, *ALL* acute lymphoblastic leukemia, *CR* complete remission, *#MA* myeloablative (according to reference 17), *GvHD* graft-versus-host disease, *ATG* antithymocyte globulin, *CyA* cyclosporine A, *MTX* metothrexate, PT-CY high dose post-transplant cyclophosphamide, *Hb* hemoglobin, *WBC* white blood cells, *PB* peripheral blood

### CMV detection

CMV infection was defined as a positive pp65 CMV antigenemia or a positive qPCR. Antigenemia was considered positive with ≥1/2.5 × 10^5^ antigen-positive cell [20], whereas qPCR was considered positive with >1000 genomes/ml. For patients with positive CMV infection, surveillance was routinely performed, as described by Marchetti and coworkers [21]. Resolution of CMV infection was defined as two consecutive negative samples. For patients diagnosed with qPCR, CMV response was defined as two consecutive negative qPCR [5]. For patients monitored with antigenemia, the resolution of CMV infection was confirmed with qPCR. Patients were monitored for viremia or antigenemia twice weekly during admission, and then at each outpatient visit for the first year, or longer in case of ongoing immunosuppressive therapy.

### CMV treatment

Treatment with foscarnet was started at first CMV positivity either with pp65 antigenemia or with PCR. Foscarnet was used as first line treatment in 62 patients (64%), all patients had at least one cytopenic cell line: 36 patients (61%) had a white blood cell (WBC) count of <1 × 10^9^/l, 40 patients (65%) had a hemoglobin (Hb) level of <9 gr/dl, and 23 patients (37%) had thrombocytopenia (<20 × 10^9^/L). Patients with persistent neutropenia <0.5 × 10^9^/L are usually treated also with G-CSF. No patient had an impaired renal function before treatment. The median daily dose of foscarnet as first line preemptive treatment was 165 mg/Kg (range 50–200). Thirty-five patients received foscarnet as second line (*n* = 32) or third line (*n* = 3) treatment because of a lack of response to previous ganciclovir/ valganciclovir treatment and/or cytopenia. The median daily dose of the second and the third line treatment was 87 mg/Kg (range 35–180) (*p* = 0.1). The dose of foscarnet for second line therapy was lower, as compared to first line therapy, possibly to reduce toxicity, but still in the range of the dose used in the prospective randomized trial (90 mg/kg) [12]. No patient had impaired renal function before starting treatment with foscarnet; leukopenia was documented in five patients (14%), thrombocytopenia in eight patients (23%) and anemia in nine patients (26%). For 26 patients, foscarnet was the only salvage therapy prescribed, whereas nine patients received foscarnet plus ganciclovir, and one patient received foscarnet plus valganciclovir. The median duration of foscarnet treatment ranged from 2 to 91 days (median 14 days) and it was similar between first and second or third lines foscarnet (median 14 vs 15 days), whereas the median duration of foscarnet was lower when used alone, rather than as combined therapy (13 vs 22, *p* = 0.05).

### Toxicity definitions

For each patient, the WBC count, neutrophils count, Hb level, platelets count, and serum creatinine levels were provided before the first dose of foscarnet and after the last one. According to the NCI-CTCAE4 [22], hematological toxicity was defined as grade 3 or higher, and renal toxicity was defined as grade 1 or higher.

### Statistical analysis

Pairwise comparisons were performed using the Fisher’s exact test for categorical variables, and the two-side Mann–Whitney *U-*test for continuous variables. Two-side Wilcoxon signed rank test was used for repeated continuous variables. The cumulative incidence (CI) of response to CMV was calculated with death as a competing event: Gray’s test was used to evaluate differences between the curves. When calculating the CI of TRM, the competing event was relapse-related death and vice versa. Survival was calculated using Kaplan–Meier product method, and the log-rank test was used to identify risk factors in univariate analysis. A multivariate logistic regression was run to identify significant variable for CMV response rate to foscarnet, whereas a multivariate Cox analysis was run to identify predictors of survival: variables included were recipient gender, disease phase, interval between HSCT and CMV infection, donor and recipient CMV serostatus, CMV load, donor type, and aGvHD occurrence. Median load was determined both for antigenemia and PCR assay at the diagnosis of CMV infection. CMV load was defined as “high” or “low” if the CMV load at diagnosis was respectively higher or lower than the median viral load in this study group, for the laboratory method of reference. Statistical analysis was performed using GraphPad Prism software v. 3.02 and NCSS 11.

## Results

Neutrophil engraftment (>0.5 × 10^9^/L) was achieved after a median interval of 17 and 18 days after HSCT in the UD and HAPLO groups (Table 2); acute GvHD grade II–IV developed in comparable proportion of patients in the two groups (Table 2).

**Table 2:** Transplant outcome, CMV infection and treatment

Number of patients	UD	HAPLO	*p*-value
	30	67	
Acute GvHD II–IV	9 (30%)	16 (24%)	0.5
Day of CMV infection	33 (3–132)	27 (1–665)	0.4
Antigenemia vs PCR	18 vs 12	58 vs 9	0.003
Duration of Foscarnet Tx	13 (2–91)	14 (2–71)	0.9
1st vs 2nd or 3th line	21 vs 9	41 vs 26	0.4
Alone vs association	27 vs 3	57 vs 10	0.5
Response to foscarnet	59% (43–81%)	84% (74–95%)	0.01
Time to CMV clearance (days)	16 (1–47)	9 (1–94)	0.1
*PB counts at CMV infections*			
WBC <1 × 10^9^/L	25 (83%)	45 (69%)	0.1
Lymphocytes ×10^9^/L^a^	0.4 (0.05–4.9)	0.9 (0–11.5)	0.05
Hb <10 gr/dl	25 (83%)	45 (69%)	0.1
Platelets <20 × 10^9^/L	11 (37%)	20 (31%)	0.5
Foscarnet 1st line	19 (63%)	39 (58%)	0.8
PB Lymph. ×10^9^/L post^b^	1.04 (0.04–6.8)	1.7 (0.05–7)	0.02
Transplant-related mortality	34% (20–56%)	22% (12–39%)	0.1
Actuarial survival at 3 years	51% (33–70%)	71% (58–84%)	0.02
*Causes of death*			*0.07*
Graft failure	1	1	
Toxicity	5 (17%)	4 (6%)	
GvHD	1	1	
Infections	2 (7%)	5 (7%)	
Relapse	5 (17%)	4 (6%)	
Follow-up days	446 (64–1808)	483 (43–1630)	0.9

The peripheral lymphocyte count is reported at the time of first^a^ and last^b^ foscarnet dose

Abbreviations as in Table 1

### CMV infection and response to foscarnet

**Fig. 1:**
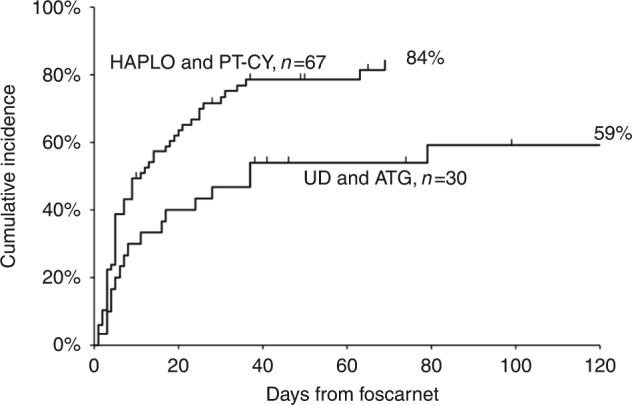
Cumulative incidence of CMV response after foscarnet according to donor type (Gray’s test, *p* = 0.01)

The median interval from HSCT to CMV infection was 33 and 27 days in UD and HAPLO (*p* = 0.4) (Table 2). The CI of response to foscarnet was overall 87%, with a median time to clearance of CMV of 7 days (range 1–79): response to foscarnet was significantly higher in patients grafted from HAPLO donor (84 vs 59%, *p* = 0.01) (Table 2, Fig. 1). Time to clear CMV was also shorter in HAPLO graft recipients (9 vs 16 days), though not significantly (*p* = 0.07) (Table 2). CI of response was higher for patients with a low viral load at the diagnosis (90.2 vs 65.8%, *p* = 0.02). PB lymphocyte counts were higher in HAPLO patients, particularly after foscarnet discontinuation (Table 2). Even though, a significantly difference in CMV response rate was not found between patients with > or <0.5 × 10^9^/L lymphocytes at the diagnosis (80 vs 64%, *p* = 0.18). The CI of response was 83% (70–98%) for first line (*n* = 62) and 92% (82–100%) for second/third line treatment (*n* = 35) with foscarnet (*p* = 0.02).

In a multivariate analysis on response, CMV load was the strongest predictor of CMV response rate to foscarnet (Table 3): patients with a lower antigenemia or viremia when starting foscarnet, had a response rate of 84%, as compared to 60% for patients with higher antigenemia or viremia (*p* = 0.02). Moreover, donor type resulted significant in univariate analysis: patients receiving HAPLO donor graft showed an higher response rate as compared to MUD (*p* = 0.02), although this was not confirmed by multivariate regression (*p* = 0.06) (Table 3).

**Table 3:** Logistic regression: CMV response rate to foscarnet

Variables	Baseline value	Compared	OR	95% CI	*p*-value
Rec serostatus	Neg	Pos	1.30	0.18–9.60	0.79
Don serostatus	Neg	pos	2.03	0.65–6.41	0.22
aGvHD^a^	No	yes	1.13	0.26–4.95	0.87
Donor	UD	HAPLO	3.46	0.96–12.43	0.06
CMV load	high	low	4.09	1.24–13.46	0.02
Gender	Female	Male	1.02	0.61–1.70	0.94
Dis. phase	Remission	Relapse	0.76	0.29–3.18	0.62
CMV onset	≤28 days	>28 days	0.41	0.11–1.55	0.19
Foscarnet line	1st	2nd/3th	0.95	0.26–2.22	0.10

^a^aGvHD at the moment of CMV infection

Abbreviations as in Table 1

An additional analysis was performed excluding the six patients for whom CMV serostatus was not available. No differences in terms of response were identified both in univariate and multivariate analysis, as compared with the reported results of the entire study group.

### Survival

**Fig. 2:**
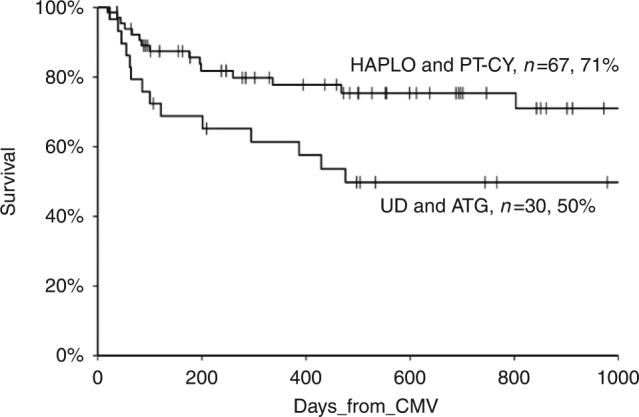
Actuarial survival after foscarnet administration for CMV infection, according to donor type (Kaplan–meier curve, log-rank test, *p* = 0.02)

After a median follow-up of 626 days (range 55–1808) from HSCT, 67 patients (69%) survive. Patients receiving a graft from a HAPLO donor with PT-Cy had a 3 year OS of 71%, compared to 50% for UD transplant with ATG (*p* = 0.02) (Fig. 2). The 3 year TRM was 34% (20–56%) for UD and 22% (12–39%) for HAPLO (*p* = 0.1) (Table 2).

In a multivariate analysis, negative factors predicting the overall survival were advanced disease phase (HR 2.92, *p* = 0.01) and ATG-based prophylaxis (HR 2.94, *P* = 0.02). Thirty patients (31%) died after a median interval of 132 days (range 43–1386). Major causes of death in the UD and HAPLO groups are shown in Table 2: graft failure (1 and 1), toxicity (5 and 4), infections (2 and 5), and relapse (5 and 4).

### Renal and hematologic toxicity

**Fig. 3:**
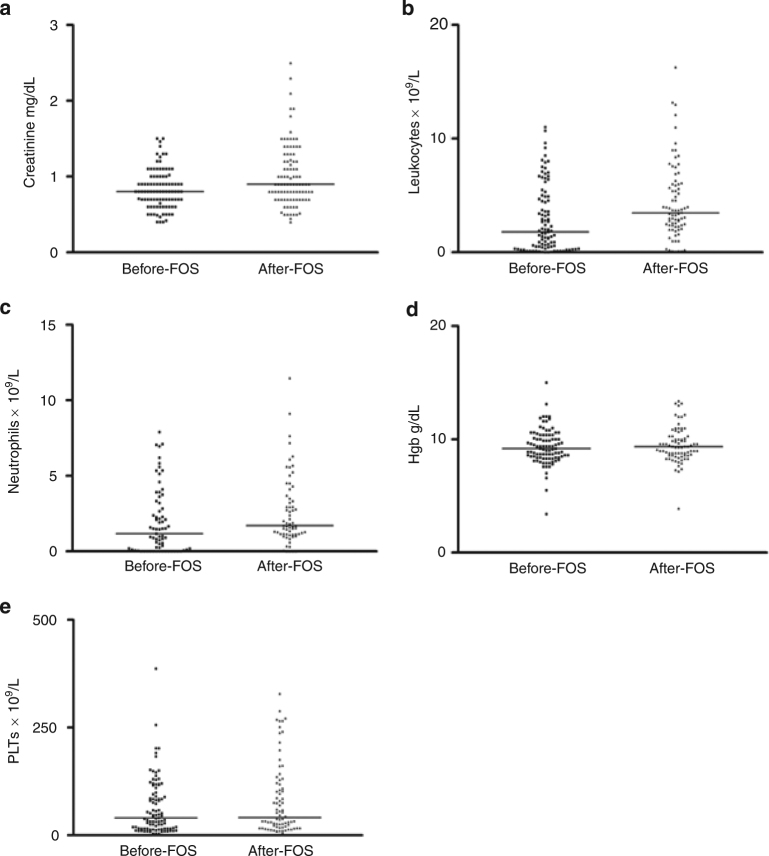
Toxicity: **a** creatinine levels before and after foscarnet; **b** leukocytes count before and after foscarnet; **c** neutrophils count before and after foscarnet; **d** hemoglobin value before and after foscarnet; **e** platelets count before and after foscarnet

The median value of creatinine was 0.8 mg/dL (range 0.4–1.5) before starting foscarnet and 0.9 mg/dL (range 0.4–2.5) after the last dose of foscarnet (*p* < 0.0001; Fig. 3a). Grade I–II renal toxicity was diagnosed in 13 patients (14%). The median number of leukocytes was 1.8 × 10^9^/L (range 0.01–11.0) before foscarnet and 3.4 × 10^9^/L (range 0.05–16.3) after foscarnet discontinuation (*p* = 0.005; Fig. 3b). Leukopenia was reported in 11 patients (14%). The neutrophils median count was 1.0 × 10^9^/L (range 0–7.9) before foscarnet and 1.7 × 10^9^/L (range 0–11.4) after foscarnet (Fig. 3c). Neutropenia was documented in 20 patients (26%). Hb data showed no difference between the level before (median 9.2 g/dL; range 5–15) and after (median 9.3 g/dL; range 5–13.4) foscarnet treatment (Fig. 3d). During foscarnet administration, anemia was recorded in six patients (8%). Finally, we did not identify a significant difference between the platelet count before (median 43.0 × 10^9^/L; range 4–527) and after (median 38.0 × 10^9^/L; range 2–329) foscarnet therapy (Fig. 3e). Thrombocytopenia was documented in 28 patients (36%). None of these abnormalities required discontinuation of foscarnet treatment (Table 4).

**Table 4:** Efficacy and toxicity of foscarnet

Patients	Overall	1st line	2nd/3th line	Single agent	Combined
	97	62	35	84	13
Therapy duration, days	14	14	15	13	22
Response, *n*	70	42	28	57	13
Time to response, days	7	8	6	7	9
*CMV viral load pp65*					
N of pos cells (median)	6	4	8	4	10
Range	(1–987)	(1–836)	(1–987)	(1–987)	(1–138)
*CMV viral load PCR*					
×10^3^ (median)	3.4	2.3	66.8	33.9	–
Range	(1.1–143)	(1.1–36.2)	(9.2–142.6)	(1.1–142.6)	–
*Foscarnet*					
Median dose (mg/Kg)	143	150	88	143	NA
Range	35–200	50–200	35–180	35–200	
Renal impairment, *n*	13 (14%)	9 (15%)	4 (11%)	12 (15%)	1 (8%)
Leukopenia, *n*	11 (14%)	6 (12%)	5 (19%)	9 (13%)	2 (22%)
Neutropenia, *n*	20 (26%)	11 (22%)	9 (33%)	16 (24%)	4 (44%)
Thrombocytopenia, *n*	28 (36%)	18 (36%)	10 (37%)	22 (32%)	6 (67%)
Anemia, *n*	6 (8%)	5 (10%)	1 (4%)	5 (7%)	1 (11%)

*NA* not available

## Discussion

We have shown in the present study that pre-emptive treatment of CMV infection with foscarnet yields higher response rates in patients grafted from HAPLO donors with PT-CY as GvHD prophylaxis, as compared to patient grafted from UD donors receiving an ATG-based regimen. We also show that renal toxicity was not a limiting factor and did not call for discontinuation.

Nephrotoxicity of foscarnet is reported to be in the range of 5 to 50% [23–26]. In the randomized foscarnet versus ganciclovir study, nephrotoxicity was comparable in the two arms: 5% in foscarnet patients versus 2% in ganciclovir patients (*p* = 0.4) [12]. In the present study, patients were treated for a relatively long period of time, with a median treatment duration of 14 days, ranging from 2 to 91 days. The median daily dose of foscarnet for first line therapy was 165 mg/kg (50–200) and for second line it was 87 mg/kg (35–180). Despite prolonged exposure to foscarnet, renal impairment grade 1–2 was diagnosed in 14% of patients, and did not cause discontinuation of foscarnet. We saw doubling of creatinine levels in two patients only (2.1%), compared to 5% of patients reported in the randomized trial foscarnet vs ganciclovir [12]: it should be pointed out that the dose of foscarnet per protocol in the randomized trial was 120 mg/kg/day. Renal toxicity could be managed in our study with appropriate hydration and electrolyte supplementation.

As to the efficacy profile, we wanted to assess the role of foscarnet in patients receiving grafts from donors other than HLA identical siblings, looking at two major strategies for GvHD prophylaxis: one based on the use of ATG in UD grafts, and the other on high dose PT-CY for HAPLO transplants. The overall rate of CMV clearance following treatment with foscarnet was 87%. In keeping with previous studies [11, 27–30], we found the CI of CMV response to foscarnet to be significantly higher in patients transplanted from a HAPLO donor with PT-CY based GvHD prophylaxis, as compared to patients transplanted from UD with ATG-based prophylaxis (84 vs 59%). However, this was not confirmed in multivariate analysis, which showed a predictive role of CMV load on the response rate. Probably, the higher response rate among HAPLO patients might be ascribed to the lower CMV load before starting foscarnet. Some authors reported an adverse impact of a high viral load on OS and TRM [31], but we did not find in the literature data regarding the effect of CMV load on response rate to antiviral agents. The Baltimore group reports a low incidence of CMV viremia, but no data on response to treatment [32]. Solomon and coworkers report 13 patients with CMV viremia, but no data on response to treatment, except for one patient who developed CMV disease [33]. When HAPLO grafts are performed with an ATG-based platform, response may be more problematic: in a pediatric study, CMV viremia developed in 24 children (41%), but CMV pneumonia was diagnosed in 7/24 (29%) [34]. No patient developed CMV pneumonia in the present study, confirming the role of foscarnet in protecting against progression of CMV infection to CMV disease [12, 13].

Immune reconstitution is a prerequisite for protection against viral infections following an allogeneic HSCT [4]: we found significantly higher lymphocyte counts in HAPLO grafts, as compared to UD patients, and this was seen before and especially after foscarnet treatment, with a median of 1.7 vs 1.0 × 10^9^/L lymphocytes in the two groups. This finding is in keeping with reports of long-lasting lymphocytopenia following ATG-based allogeneic HSCT: in a previous study [35], we have shown significantly lower CD4 counts on day +100, for UD grafts with ATG (100/μl) compared to HAPLO grafts with PT-CY (200/μl), and the difference was maintained also at 6 months post-transplant [35]. Therefore, faster immune recovery in HAPLO grafts may explain the significantly higher response rate we have seen in HAPLO transplants, once CMV infections develops and is treated preemptively. The overall survival was 69%, with a 3 year TRM of 26% and a 3 year relapse-related death of 10%, in keeping with survival reported in the literature [11, 27–30]. Again, survival appeared higher for patients receiving HAPLO donor than for patients transplanted from UD (71 vs 50%), and this data was also confirmed by multivariate analysis, which showed ATG-based prophylaxis regimen and advance disease phase at transplant, the only two significant risk factor.

In conclusion, we find significantly different response to preemptive treatment of CMV, when comparing UD grafts receiving ATG-based prophylaxis with HAPLO transplants given high dose PT-CY. GvHD prophylaxis remains predictive also on survival, together with disease phase.

Obviously, we cannot assert that the difference in response to foscarnet between the HAPLO and the UD groups is surely ascribable to donor type. In fact, other differences are recognizable among the two groups: all the patients transplanted from UD received ATG as part of GvHD prophylaxis, and we cannot exclude the role of ATG in the response rate, as well as its role in overall survival; patients transplanted from a HAPLO donor showed a faster lymphocytes recovery, as we reported previously, and also this condition might affect the best response rate, as compared to transplant from UD.
